# Physiological and transcriptome analyses of photosynthesis and chlorophyll metabolism in variegated *Citrus* (*Shiranuhi* and *Huangguogan*) seedlings

**DOI:** 10.1038/s41598-019-52276-5

**Published:** 2019-10-30

**Authors:** Bo Xiong, Xia Qiu, Shengjia Huang, Xiaojia Wang, Xu Zhang, Tiantian Dong, Tie Wang, Sichen Li, Guochao Sun, Jin Zhu, Zhihui Wang

**Affiliations:** 1 College of Horticulture, Sichuan Agricultural University, Chengdu, 611130 China; 2 Institute of Pomology and Olericulture, Sichuan Agricultural University, Chengdu, 611130 China; 3 Sichuan Horticultural Crop Extension Station, Chengdu, 610041 China

**Keywords:** Plant biotechnology, Plant sciences

## Abstract

*Citrus* species are among the most economically important fruit crops. Physiological characteristics and molecular mechanisms associated with de-etiolation have been partially revealed. However, little is known about the mechanisms controlling the expression and function of genes associated with photosynthesis and chlorophyll biosynthesis in variegated citrus seedlings. The lower biomass, chlorophyll contents, and photosynthetic parameter values recorded for the variegated seedlings suggested that chlorophyll biosynthesis was partially inhibited. Additionally, roots of the variegated seedlings were longer than the roots of green seedlings. We obtained 567.07 million clean reads and 85.05 Gb of RNA-sequencing data, with more than 94.19% of the reads having a quality score of Q30 (sequencing error rate = 0.1%). Furthermore, we detected 4,786 and 7,007 differentially expressed genes (DEGs) between variegated and green *Shiranuhi* and *Huangguogan* seedlings. Thirty common pathways were differentially regulated, including pathways related to photosynthesis (GO: 0015979) and the chloroplast (GO: 0009507). Photosynthesis (44 and 63 DEGs), photosynthesis-antenna proteins (14 and 29 DEGs), and flavonoid biosynthesis (16 and 29 DEGs) pathways were the most common KEGG pathways detected in two analyzed libraries. Differences in the expression patterns of *PsbQ*, *PetF*, *PetB*, *PsaA*, *PsaN*, *PsbP*, *PsaF*, Cluster-2274.8338 (*ZIP1*), Cluster-2274.38688 (*PTC52*), and Cluster-2274.78784 might be responsible for the variegation in citrus seedlings. We completed a physiological- and transcriptome-level comparison of the *Shiranuhi* and *Huangguogan* cultivars that differ in terms of seedling variegation. We performed mRNA-seq analyses of variegated and green *Shiranuhi* and *Huangguogan* seedlings to explore the genes and regulatory pathways involved in the inhibition of chlorophyll biosynthesis and decreases in Chl *a* and Chl *b* contents. The candidate genes described herein should be investigated in greater detail to further characterize variegated citrus seedlings.

## Introduction

*Citrus*, which have been cultivated globally, is one of the most important fruit species^1^. *Shiranuhi* [*Citrus reticulata* × (*Citrus reticulata* × *Citrus sinensis*)] and *Huangguogan* (*Citrus reticulata* × *Citrus sinensis*) are relatively new hybrid citrus cultivars grown in China^2^. Etiolation, which occurs in many angiosperms, refers to the phenomenon that leaves appear yellow under dark conditions. Seedlings undergo skotomorphogenesis in lightless culture condition, and leaves take on the color of carotenoids. This phenomenon results in a rapid elongation of the hypocotyl^3^. Changes in plant morphology and growth are the ultimate reflection of plant damages caused by etiolation. This phenomenon leads to decreased leaf and optical areas, the production of dwarf plants, a weakened growth potential, and even death. A stable supply of chlorophyll, which is the main photosynthetic pigment of plants, is a key requirement for the normal development of photosynthesis^4^. Analyses of light regulation^5^, ethylene responses^6^, riboflavin biosynthesis^7^, endogenous abscisic acid levels^3^, phospholipid hydroperoxide glutathione peroxidase activity^8^, and proteomics analysis^9^ have been used to examine plant growth and development induced by etiolation. Many studies have focused on the expression analysis of related genes during photosynthesis in greening, and the regulation of the corresponding protein levels^10^. However, there have been relatively few transcriptome-level studies of variegation in citrus seedlings.

Variegated plants, which have both green and white or yellow areas on the same leaf, are considered as the invaluable materials for studying chloroplast biosynthesis, development and maintenance^11^. The white sectors of var2, which is one of the Arabidopsis (*Arabidopsis thaliana*) mutants showing leaf variegation, are active tissues that are formed by viable cells with undifferentiated plastids^12^. Chloroplast development and chlorophyll accumulation are inhibited at the albinism growth stage of albino tea cultivars^13^. In photosynthetic organisms, chlorophyll and carotenoid are the main pigments of plants that capture light energy. Earlier studies revealed that the etiolated growth of seedlings or leaves considerably affects chloroplast development and chlorophyll metabolism^14–16^. Whether the seedlings undergo etiolation or exhibit variegation, both lead to color changes of leaves.

Next-generation sequencing (NGS) technology is a rapid and cost-effective approach to analyzing a large number of protein-coding genes^17,18^. This technology can be used to address questions related to ecological comparative and evolutionary genomics in non-model organisms^19^. Based on the similar expression pattern, using transcriptomic data to group differentially expressed genes (DEGs) is one of the most effective methods to explore the relationships and predict functions of candidate genes^20,21^. Similarly expressed genes are often functionally related^22^. In the present study, we identified variegation -related genes and examined the pathways associated with photosynthesis and chlorophyll biosynthesis using RNA-sequencing (RNA-seq) combined with the analysis of chlorophyll contents to characterize the variegation in citrus seedlings. Our findings may help clarify the molecular basis of variegation.

We recently analyzed the transcriptome of etiolated citrus seedlings, there were variegated and green seedlings in both *Shiranuhi* and *Huangguogan* under the same conditions of seedling germination^23^. Although variegated plants exist widely in nature, the mechanism of citrus leaf variegation and the mechanisms regulating the expression patterns of well-characterized genes involved in photosynthesis and chlorophyll biosynthesis are still unknown. In order to study the color difference of citrus leaf after germination under the same culture condition, transcriptome sequencing was performed on the leaves of citrus seedlings. In the present study, we compared the leaf biomass and chlorophyll contents of variegated and green *Shiranuhi* and *Huangguogan* seedlings. In addition to the differentially expressed photosynthesis-related genes, the expression profiles of chlorophyll biosynthesis-related genes were analyzed to reveal the molecular mechanism underlying variegation.

## Results

### Biomass accumulation of variegated and green seedlings

**Figure 1:**
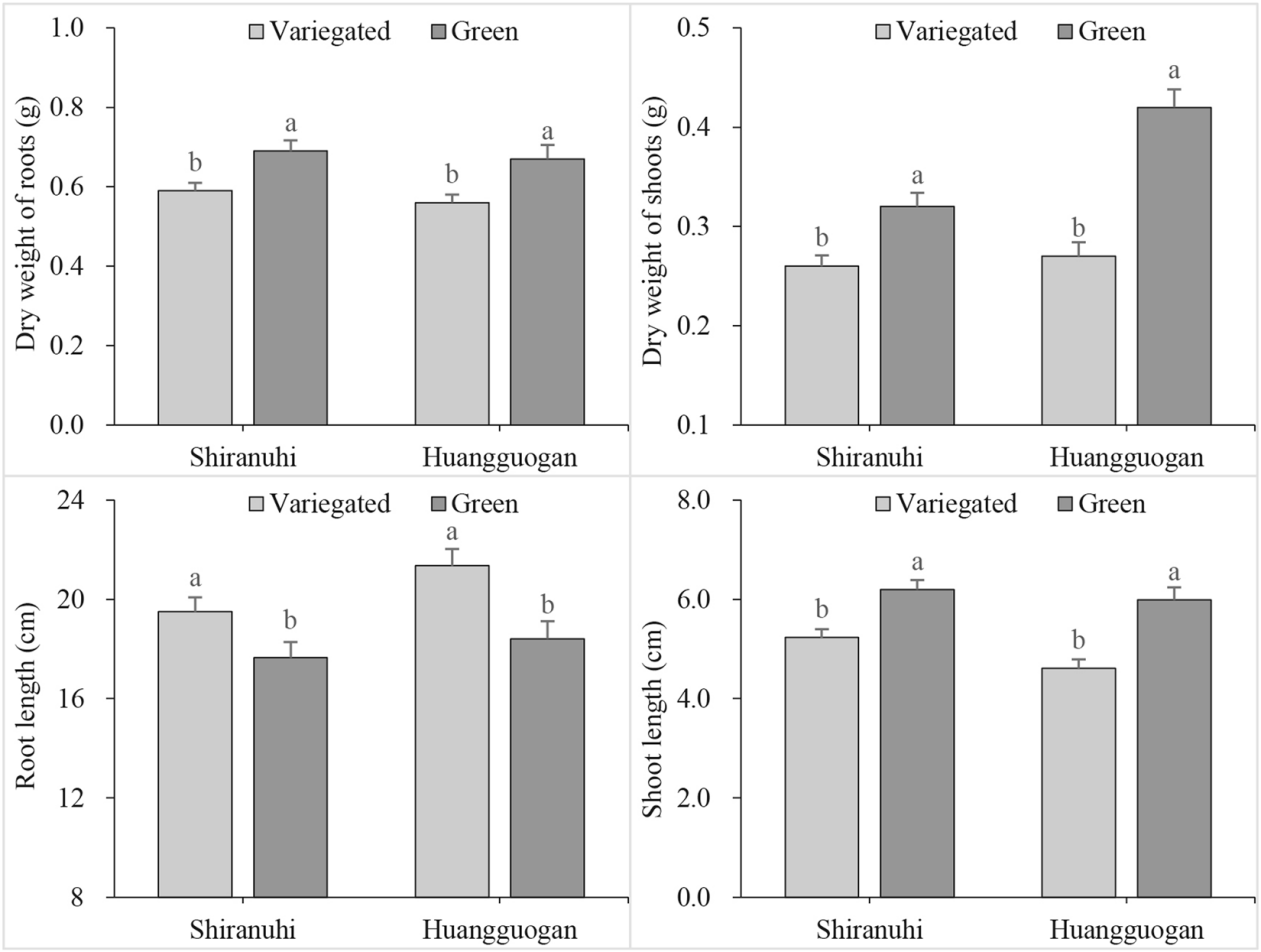
Effect of etiolation on growth of variegated and green seedlings of *Huangguogan* and *Shiranuhi*.

Variegated and green *Shiranuhi* and *Huangguogan* seedlings were sampled at 20 days after germination to measure dry weight, length, and chlorophyll contents. We observed a significant difference in shoot and root dry weight between the variegated and green seedlings. Additionally, the roots of variegated seedlings were longer than those of green seedlings for both analyzed cultivars, while the opposite trend was observed for the shoots. Furthermore, the shoot lengths of green seedlings were longer than those of variegated seedlings in both *Shiranuhi* and *Huangguogan*, while the roots were shorter (Fig. 1). These results indicated that the variegation of citrus leaves had different effects on seedling root and shoot lengths.

### Chlorophyll content and photosynthesis of variegated seedlings

**Figure 2:**
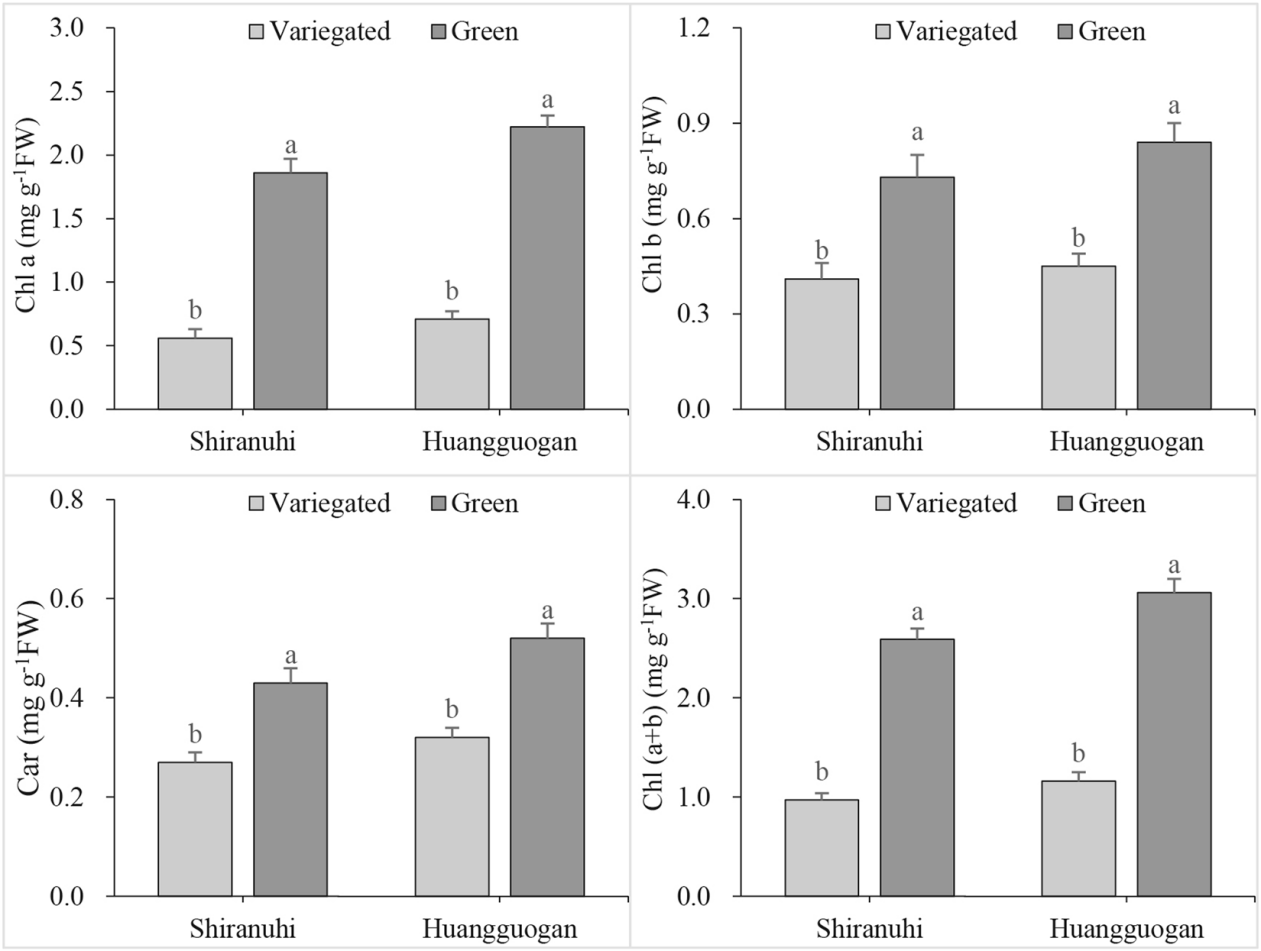
Effect of etiolation on the photosynthetic pigment contents of variegated and green seedlings of *Huangguogan* and *Shiranuhi*.

The chlorophyll *a* (Chl *a*) concentration of variegated *Shiranuhi* and *Huangguogan* seedlings were 0.56 and 0.71 mg g^−1^ fresh weight, respectively (Fig. 2), while the corresponding concentrations in green seedlings were 1.86 and 2.22 mg g^−1^ fresh weight, respectively. Similarly, chlorophyll *b* (Chl *b*) and carotenoid contents as well as the composition and proportion of photosynthetic pigments exhibited the same trends as the Chl *a* content.

**Figure 3:**
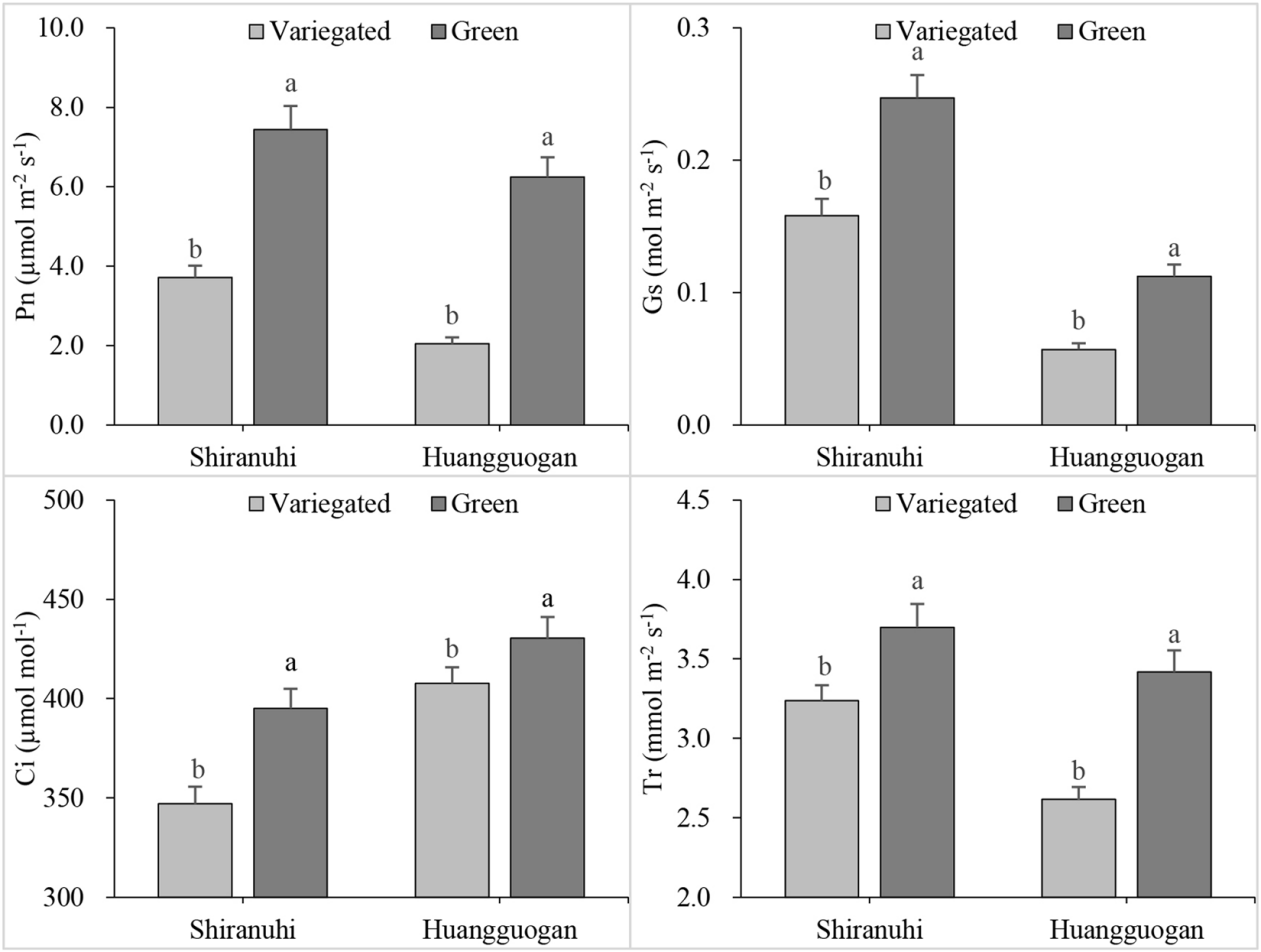
Photosynthetic gas exchange parameters of variegated and green seedlings of *Huangguogan* and *Shiranuhi*.

The photosynthetic parameters differed between the *Huangguogan* and *Shiranuhi* variegated and green seedlings (Fig. 3). For example, the net photosynthetic rate (Pn), intercellular CO_2_ concentration (Ci), and transpiration rate (Tr) were lower in variegated seedlings than in green seedlings. Additionally, the photosynthetic parameter values were lower for *Huangguogan* than for *Shiranuhi* seedlings, except for the Ci.

### Sequencing and assembly of expressed genes

To investigate the genome-wide expression patterns of variegated seedlings, more than 10 leaves harvested from each seedling type for each cultivar at 20 days after germination were analyzed by RNA-seq. We constructed four cDNA libraries, namely R_V and R_G, which represent variegated and green *Shiranuhi* seedlings, respectively, and Y_V and Y_G, which correspond to variegated and green *Huangguogan* seedlings, respectively. After removing the sequencing adapters and low-quality data, we obtained 567.07 million clean reads and 85.05 Gb of RNA-seq data. More than 94.19% of the reads had a quality score of Q30 (sequencing error rate = 0.1%). The sequencing data statistics are listed in Table 1. The raw data were deposited in the NCBI Gene Expression Omnibus database (accession number GSE90935).

**Table 1:** Overview of the sequencing results.

Sample	Raw Reads	Clean Reads	Clean Bases (G)	Q30 (%)	GC Content (%)	Mapped Ratio^a^ (%)
R_G_1	57012302	54988870	8.25	94.42	43.89	83.42
R_G_2	41815870	41233266	6.18	94.60	44.18	84.78
R_G_3	41604600	40730430	6.11	94.60	44.26	83.95
R_V_1	48582150	46739478	7.01	94.29	44.12	83.44
R_V_2	56099636	53936088	8.09	94.19	44.18	83.19
R_V_3	50374534	48399808	7.26	94.30	44.28	83.12
Y_G_1	47891158	45978328	6.90	94.36	44.28	83.43
Y_G_2	47860120	46055110	6.91	94.47	44.18	83.91
Y_G_3	54082116	52015908	7.80	94.38	44.19	83.10
Y_V_1	47357694	45747250	6.86	94.48	44.41	85.39
Y_V_2	49140048	47483244	7.12	94.57	44.43	85.38
Y_V_3	45257470	43761800	6.56	94.75	44.43	85.45

R_G, *Shiranuhi* green seedlings; R_V, *Shiranuhi* variegated seedlings; Y_G, *Huangguogan* green seedlings; Y_V, *Huangguogan* variegated seedlings. ^a^Percentage of clean reads that were mapped to transcripts or unigenes.

### Evaluation of gene expression levels

**Figure 4:**
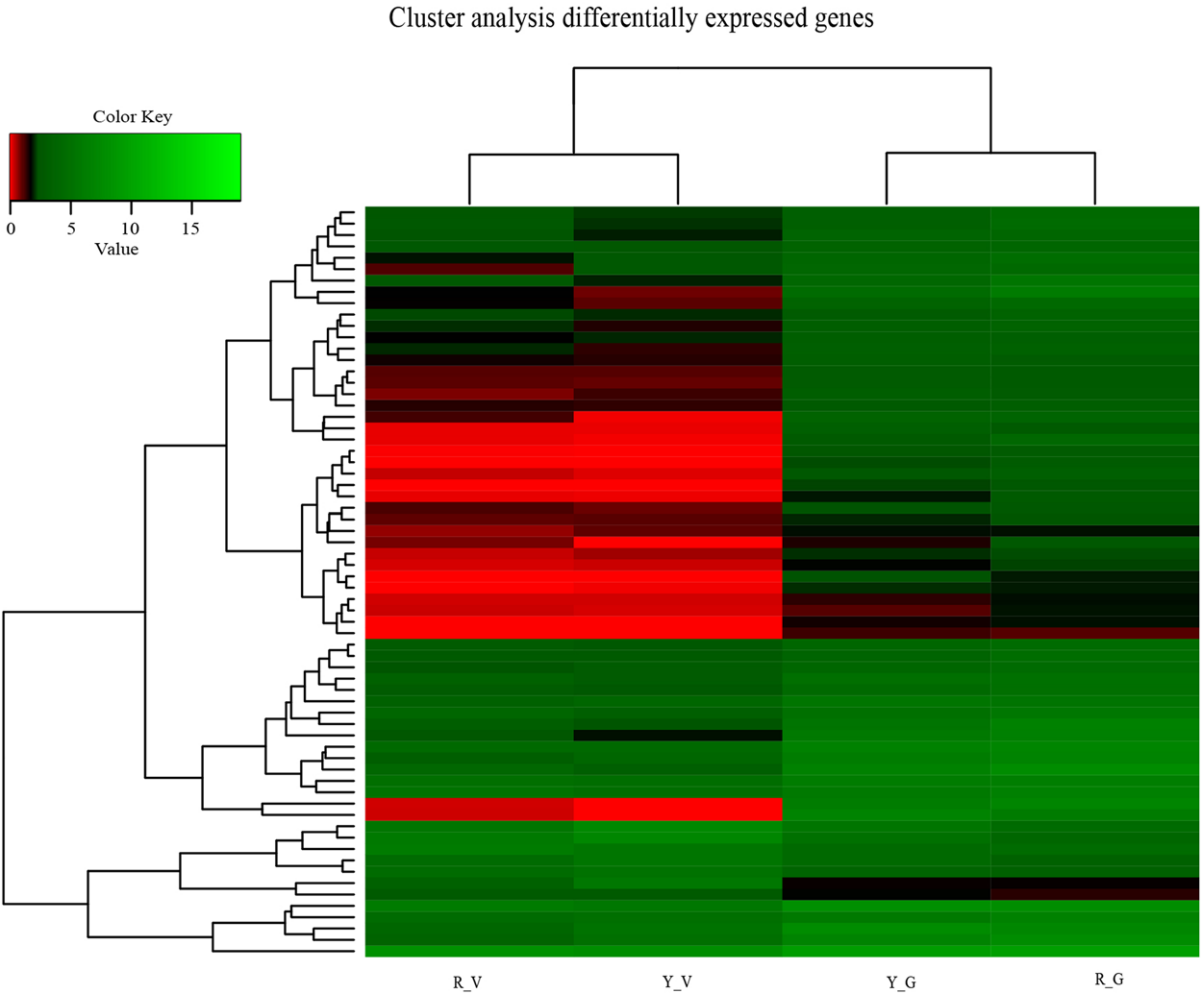
Hierarchical cluster analysis of DEGs in four libraries. Hierarchical cluster analysis was carried out with 66 significantly differentially expressed genes in green and variegated seedlings of *Shiranuhi* and *Huangguogan*. Value = log_2_ FPKM.

The number of fragments per kilobase of exon per million fragments mapped (FPKM) was used to quantify the unigene expression levels, which were detected by RNA-seq with high sensitivity. Hierarchical cluster analyses were conducted with 66 DEGs identified for variegated and green *Shiranuhi* and *Huangguogan* seedlings. Genes with the same or similar expression profiles were clustered to present the gene sets exhibiting different expression patterns under various experimental conditions (Fig. 4).

### Identification and functional annotation of differentially expressed genes

**Figure 5:**
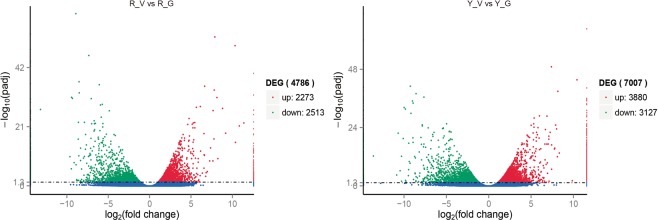
Differential expression analysis of unigenes in the variegated seedlings (M) versus the green seedlings (G) of *Shiranuhi* (R) and *Huangguogan* (Y). The log_2_(fold change) is used as the x-axis and −log_10_(pdaj) is used as the y-axis. 2273 and 3880 unigenes were up-regulated in R_V and Y_V (highlighted in green), and 2513 and 3127 unigenes were down-regulated (highlighted in red), respectively.

**Figure 6:**
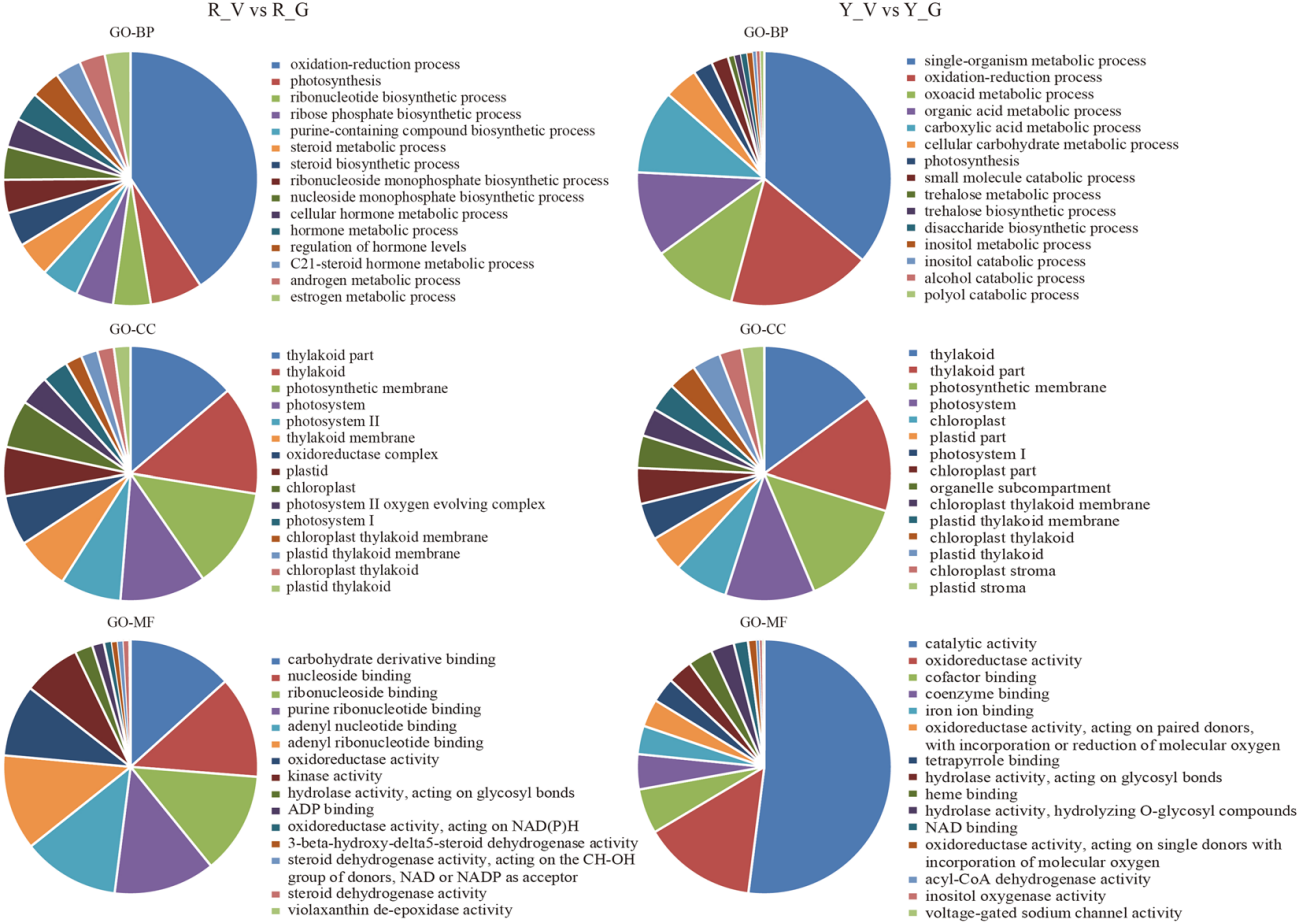
Gene Ontology functional classification analysis of genes with different expression patterns of variegated seedlings of *Shiranuhi* and *Huangguogan* (*P*-value < 0.01). Gene ontology biological process (GO-BP), gene ontology cellular component (GO-CC), and gene ontology molecular function (GO-MF).

During the screening of DEGs, we used *P* value < 0.005^24^ and |log2 (fold change)| > 1 as the threshold criteria to determine the significance of the differences in gene expression levels. A total of 4,786 and 7,007 DEGs between variegated and green *Shiranuhi* and *Huangguogan* seedlings were classified into the gene ontology (GO) categories biological process (GO-BP), molecular function (GO-MF), and cellular component (GO-CC) (Figs 5–6). For the R_V library, significant differences in the enrichment of 30 biological processes were detected in the GO-BP class, including photosynthesis (79 DEGs), cellular hormone metabolic process (44 DEGs), hormone metabolic process (44 DEGs), regulation of hormone levels (44 DEGs), oxidation-reduction process (485 DEGs), and other processes. Additionally, ADP binding (79 DEGs), oxidoreductase activity (482 DEGs), 3-beta-hydroxy-delta 5-steroid dehydrogenase activity (39 DEGs), and 19 other processes were significantly enriched in the GO-MF class. Furthermore, photosystem (72 DEGs), photosystem II oxygen-evolving complex (25 DEGs), chloroplast (40 DEGs), chloroplast thylakoid membrane (14 DEGs), chloroplast thylakoid (14 DEGs), photosystem II (51 DEGs), and photosystem I (22 DEGs) were significantly enriched in the GO-CC class (Fig. 6). For the Y_V library, significant differences in enrichment were observed for 83 sub-categories in the GO-BP class, 28 in the GO-MF class, and 32 in the GO-CC class. A comparison of both libraries indicated that 15 sub-categories were related to the GO-BP class, such as oxidation-reduction process (GO: 0055114), single-organism metabolic process (GO: 0044710), and photosynthesis (GO: 0015979). Meanwhile, 13 sub-categories were related to the GO-CC class, such as photosystem I (GO: 0009522), chloroplast thylakoid (GO: 0009534), chloroplast thylakoid membrane (GO: 0009535), photosynthetic membrane (GO: 0034357), photosystem (GO: 0009521), and chloroplast (GO: 0009507). In contrast, only oxidoreductase activity (GO: 0016491) was related to the GO-MF class (Fig. 6).

**Figure 7:**
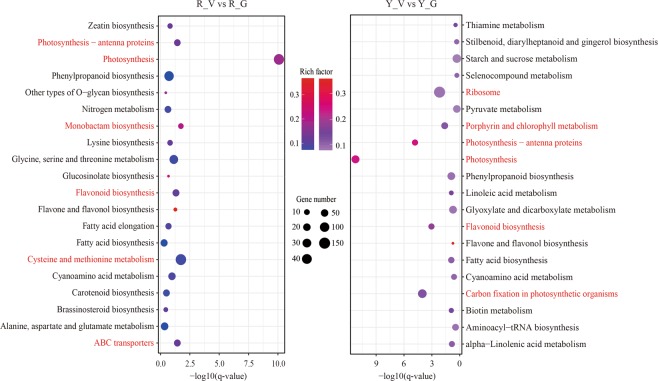
Statistics of the top 20 KEGG pathway enriched with genes with different expression patterns of variegated seedlings of *Shiranuhi* and *Huangguogan*.

To investigate the biological pathways important for the variegation in *Shiranuhi* and *Huangguogan* seedlings, the DEGs between variegated and green seedlings were further annotated based on the reference pathways in the Kyoto Encyclopedia of Genes and Genomes (KEGG) database (Fig. 7). These DEGs in the R_V and Y_V libraries were assigned to 117 and 119 KEGG pathways, respectively. The pathway search results were sorted based on the number of hits, and photosynthesis (44 and 63 DEGs), photosynthesis-antenna proteins (14 and 29 DEGs), and flavonoid biosynthesis (16 and 29 DEGs) were the most highly represented pathways in both libraries. Genes associated with cysteine and methionine metabolism (47 DEGs), monobactam biosynthesis (9 DEGs), and ABC transporters (15 DEGs) were also common in the R_V library (Fig. 7). In contrast, genes related to carbon fixation in photosynthetic organisms (80 DEGs), ribosome (154 DEGs), and porphyrin and chlorophyll metabolism (41 DEGs) were common in the Y_V library (*P* value < 0.05).

### Analysis of chlorophyll biosynthesis and photosynthesis-related transcripts

**Figure 8:**
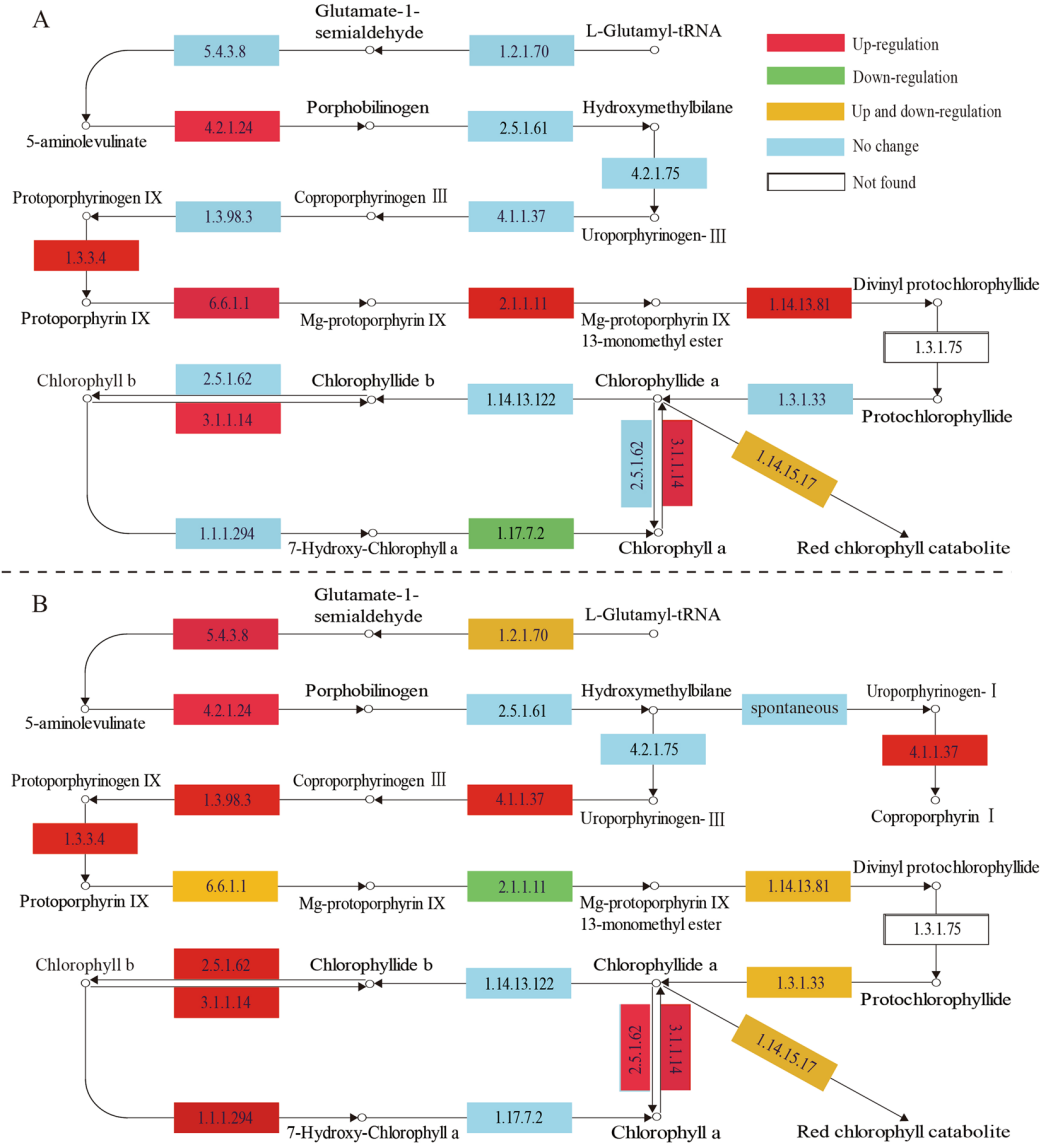
Chlorophyll metabolism-related gene expression-level changes in the chlorophyll biosynthesis pathways of (**A**) *Shiranuhi* and (**B**) *Huangguogan* seedlings. Numbers in boxes correspond to the following EC numbers: 4.2.1.24, porphobilinogen synthase. 2.5.1.61, hydroxymethylbilane synthase. 4.1.1.37, uroporphyrinogen decarboxylase. 4.2.1.75, uroporphyrinogen-III synthase. 4.1.1.37, uroporphyrinogen decarboxylase. 1.3.98.3, oxygen-independent coproporphyrinogen III oxidase. 1.3.3.4, protoporphyrinogen oxidase. 6.6.1.1, magnesium chelatase subunit H, D, I. 2.1.1.11, magnesium-protoporphyrin O-methyltransferase. 1.14.13.81, magnesium- protoporphyrin IX monomethyl ester (oxidative) cyclase. 1.3.1.75, divinyl chlorophyllide *a* 8-vinyl-reductase. 1.3.1.33, protochlorophyllide reductase. 2.5.1.62, chlorophyll synthase. 3.1.1.14, chlorophyllase. 1.14.13.122, chlorophyllide *a* oxygenase. 1.1.1.294, chlorophyll(ide) *b* reductase. 1.17.7.2, 7-hydroxymethyl chlorophyll *a* reductase.

Chlorophyll is a key photosynthetic pigment in plant chloroplasts, and chlorophyll metabolism is an important factor influencing crop yield^25^. To investigate whether photosynthesis pathway genes are involved in this decrease, we analyzed the expression patterns of genes encoding regulatory enzymes associated with chlorophyll biosynthesis (Fig. 8). Fifteen enzymes are required for the chlorophyll biosynthesis from glutamyl-tRNA to Chl *b*^26^. Magnesium (Mg) chelation is the first step in the cholorophyll branch of porphyin metabolism, forming magnesium-protoporphyrin (MgP, EC: 2.1.1.11), which is followed by a SAM-dependent methylation of the 6-propionate side chain to form Mg-protoporphyrin monomethyl ester (MgPME)^27^. We observed that the expression of the gene encoding magnesium chelatase subunit I (MgCE I, EC: 6.6.1.1) was down-regulated from 580.67 to 15.05 FPKM (Cluster-2274.70486) in the Y_V library. Magnesium-protoporphyrin IX monomethyl ester (oxidative) cyclase (MgPMEC, EC: 1.14.13.81) is another key enzyme involved in chlorophyll biosynthesis. Our RNA-seq data revealed a considerable difference in the abundance of *MgPMEC* transcripts (Cluster-2274.8338), with 46.77 FPKM in the Y_G library and 0 FPKM in the Y_V library, this was consistent with previous research about variegated *Epipremnum aureum*^11^. Meanwhile, protochlorophyllide reductase (POR, EC: 1.3.1.33) catalyzes a reaction with divinyl-protochlorophyllide as a substrate to synthesize divinyl-chlorophyllide *a*^25^. The expression levels of the gene encoding POR (Cluster-2274.3601) were down-regulated, with 19.73 FPKM in the Y_G library and 0 FPKM in the Y_V library. Furthermore, in the chlorophyll biosynthesis pathway, 7-hydroxymethyl chlorophyll *a* reductase (HCAR, EC: 1.17.7.2) converts Chl *b* to 7-hydroxymethyl Chl *a* and ultimately to Chl *a*^28^. We observed no changes in the expression levels of the gene encoding HCAR in the Y_V library, but it was down-regulated from 51.14 to 10.30 FPKM (Cluster-2274.20724) in the R_V library.

In comparisons between the R_V and R_G libraries and between the Y_V and Y_G libraries, we detected six common DEGs related to porphyrin and chlorophyll metabolism, namely Cluster-2274.78784 (K08099, 3.1.1.14), Cluster-2274.38688 (K13071, 1.14.15.17), Cluster-2274.8338 (K04035, 1.14.13.81), Cluster-2274.66782 (K03403, 6.6.1.1), Cluster-2274.56424 (K13071, 1.14.15.17), and Cluster-2274.70486 (K03428, 2.1.1.11) (Fig. 8). The comparison between the R_V and R_G libraries revealed 44 DEGs related to the photosynthesis pathway, including 13 genes related to photosystem I (PSI), 15 related to photosystem II (PSII), four related to the cytochrome b6/f complex, three related to photosynthetic electron transport, and nine related to the F-type ATPase. In contrast, the comparison between the Y_V and Y_G libraries detected 63 DEGs associated with the photosynthesis pathway, including 26, 15, 5, 10, and 7 related to PSI, PSII, cytochrome b6/f complex, photosynthetic electron transport, and F-type ATPase, respectively.

**Figure 9:**
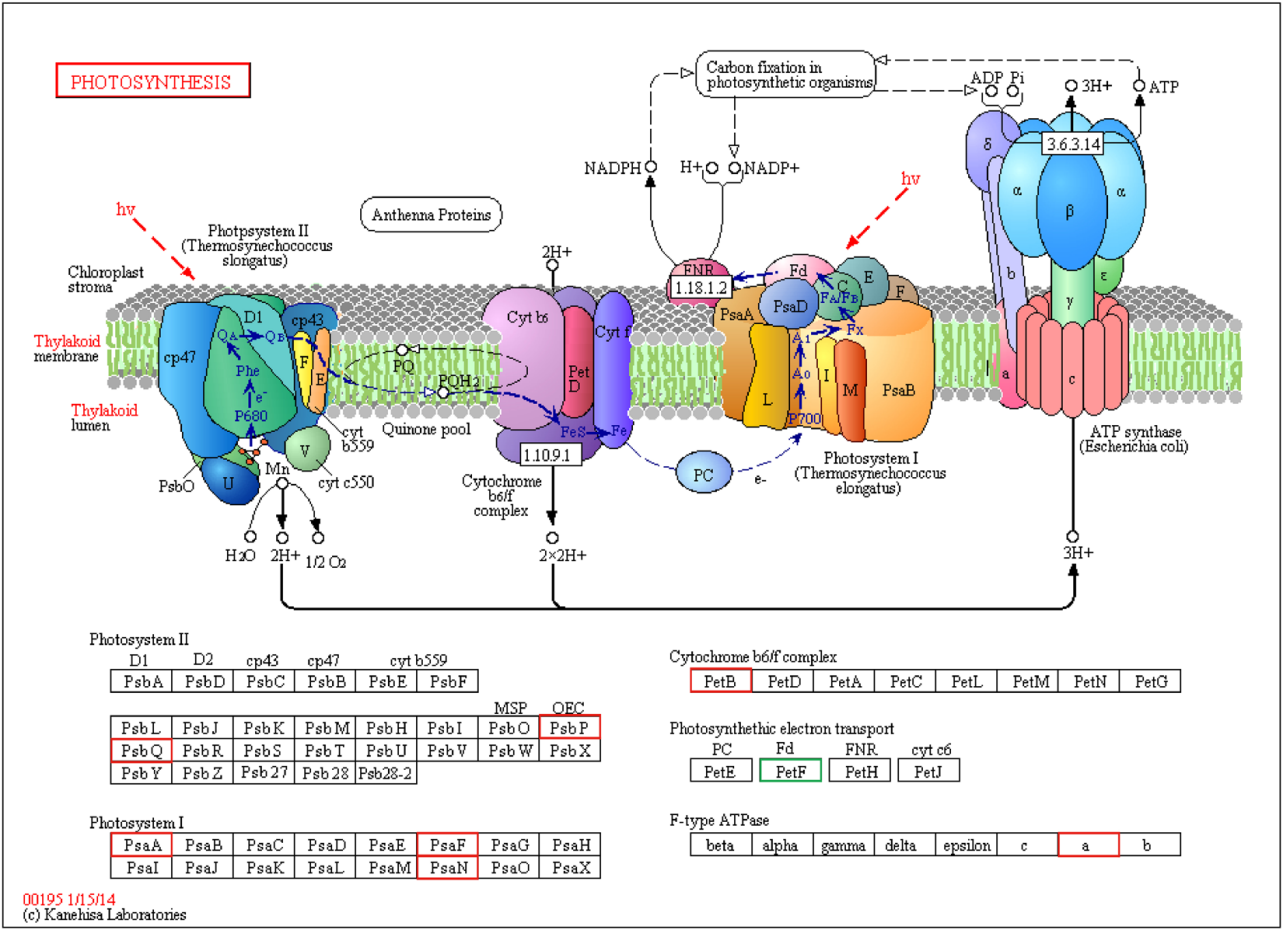
Differentially expressed genes mapped to photosynthesis pathway. The known pathways were obtained from the KEGG database. Red square denotes up-regulated expression in variegated seedlings of *Shiranuhi* and *Huangguogan* compared with that of green seedlings, respectively, while green square denotes both up- and down-regulated genes. We have got the formal permission of KEGG imagery (map00195)^29,30^ from Kanehisa laboratories.

In comparisons between the R_V and Y_V libraries and between the R_G and Y_G libraries, the following nine common DEGs associated with the photosynthesis pathway were identified: Cluster-2274.72645 (*PsbQ*), Cluster-2274.39503 (*PetF*), Cluster-2274.81464 (*PetF*), Cluster-2274.51835 (*PetB*), Cluster-2274.57179 (F-type ATPase a), Cluster-2274.57012 (*PsaA*), Cluster-19856.1 (*PsaN*), Cluster-17851.0 (*PsbP*), and Cluster-2274.55964 (*PsaF*), and these DEGs were marked on KEGG imagery (map00195) from Kanehisa laboratories^29,30^ (Fig. 9). Additionally, 15 genes involved in photosynthetic activities and chlorophyll biosynthesis may be associated with the differences in leaf color between the R_V and Y_V seedlings (Figs 8,9).

### qRT-PCR validation

**Figure 10:**
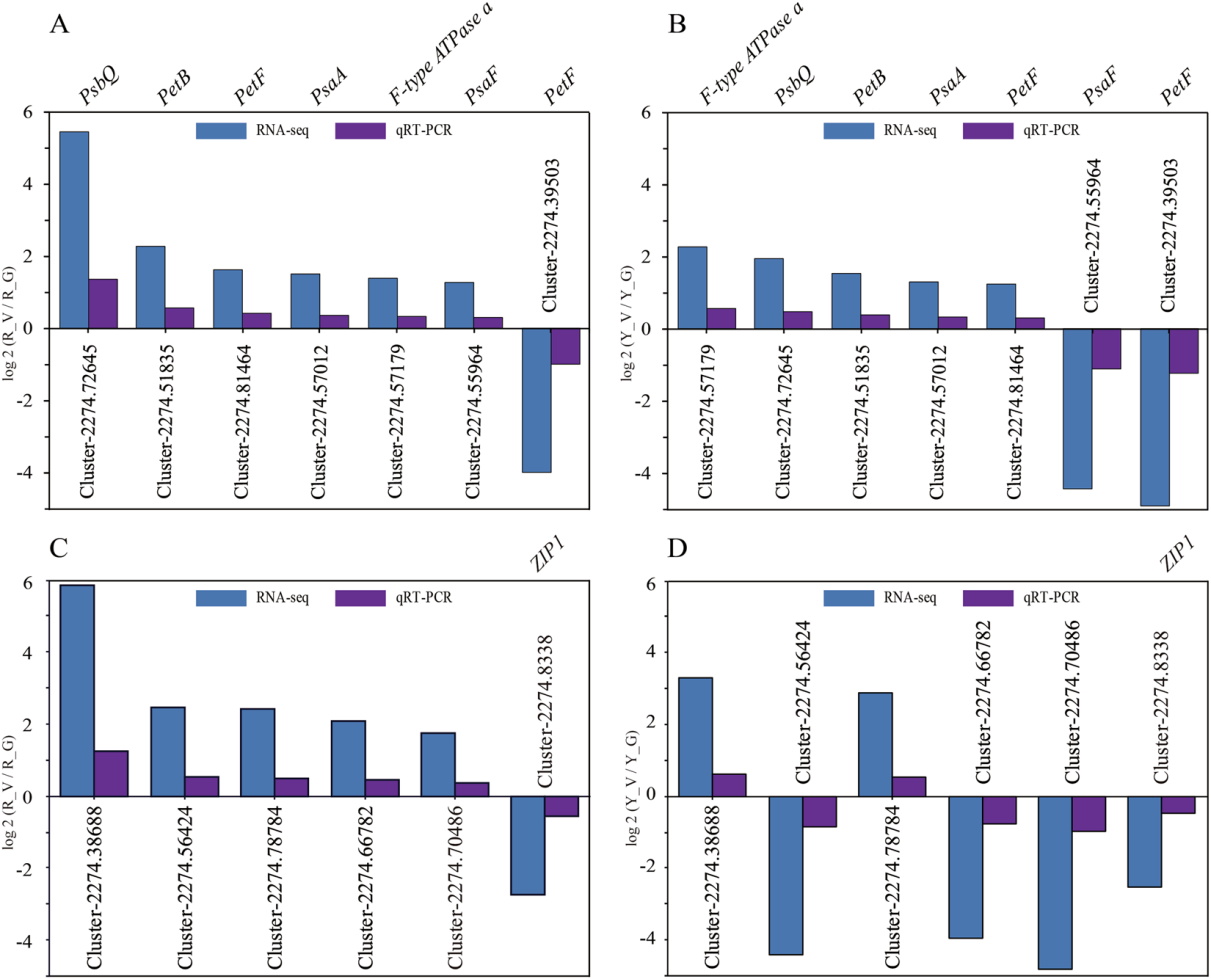
Expression pattern of 7 photosynthesis-related and 6 chlorophyll metabolism-related genes as obtained by RNA-seq and qRT-PCR. (**A**) qRT-PCR validation for the 7 photosynthesis-related genes of *Shiranuhi*. (**B**) qRT-PCR validation for the 7 photosynthesis-related genes of *Huangguogan*. (**C**) qRT-PCR validation for the 6 chlorophyll metabolism-related genes of *Shiranuhi*. (**D**) qRT-PCR validation for the 6 chlorophyll metabolism-related genes of *Huangguogan*. R_V, R_G, Y_V and Y_G represent normalized expression levels for the DEGs in the *Shiranuhi* and *Huangguogan* libraries, respectively. FC is the ratio of FPKM between variegated and green seedlings.

We selected photosynthetic genes (*PsbQ*, *PetF*, *PetB*, *PsaA*, *PsaN*, *PsbP*, and *PsaF*) and 6 porphyrin and chlorophyll metabolism pathway genes from common DEGs in R_V and Y_V libraries and evaluated their expression profiles using quantitative real-time PCR. *Actin* (GenBank: XM 006480741.2) was selected for internal controls. The verification results of the 7 photosynthesis-related and 6 chlorophyll metabolism-related DEGs demonstrated that nearly all of the common photosynthetic genes showed similar expression patterns of RNA-seq analysis (Fig. 10). In the porphyrin and chlorophyll metabolism pathway, there were three common DEGs (Cluster-2274.8338, Cluster-2274.38688, Cluster-2274.78784) with a similar expression pattern in R_V and Y_V seedlings. The other three common DEGs showed the opposite expression pattern, up-regulated in R_V and down-regulated in Y_V (Fig. 10).

## Discussion

The purpose of this research was to study the differences in the transcriptome expression of variegated and green *Huangguogan* and *Shiranuhi* seedlings, especially regarding photosynthesis and chlorophyll biosynthesis. We identified major transcriptomic features in the variegated seedlings.

Photosynthetic activities are affected by stomatal conductance (Gs), Ci, Tr, and chlorophyll fluorescence^31^. The Pn directly reflects the photosynthetic capacity^32^, while Ci is an important indicator of stomatal activity. During photosynthesis, a decrease in Tr caused by a decrease in Gs leads to a significant decrease in transpiration, which can inhibit the absorption and transport of water and nutrients^33^. In addition to shorter roots, the variegated seedlings also had significantly lower seedling biomass, chlorophyll content, and photosynthetic parameter values (*P* < 0.05) compared with the green seedlings (Figs 1–3). The identification and functional annotation of DEGs indicated that the photosynthesis pathway genes (44 and 63 DEGs) were the most common genes in the R_V and Y_V libraries (based on the number of hits for DEGs), with the lowest corrected *P* values (8.17212942029e-11 and 6.07238386484e-12) (Fig. 7).

Transcriptional regulation is important for coordinating the conversion of glutamyl-tRNA to the final product (Chl *a* and Chl *b*). There are 15 enzymes encoded by 27 genes in *Arabidopsis thaliana* in chlorophyll synthesis^4^. The six-electron oxidation of protogen IX is catalyzed to protoporphyrin IX (proto IX) by Protoporphyrinogen IX (protogen IX) oxidase (PPOX), with flavin as the cofactor^34^. Previous studies revealed that MgPMT (Mg-protoporphyrin IX methyltransferase) activity is inversely related to the transcriptional activities associated with Mg-chelatase and ALA synthesis, and restricts the formation of chlorophyll^4,35^. Low expression levels of Cluster-2274.8338 (K04035, 1.14.13.81) lead to a decrease in divinyl protochlorophyllide synthesis, while the up-regulated expression of Cluster-2274.78784 (K08099, 3.1.1.14) induces the transformation of Chl *a* to Chlorophyllide *a*. Furthermore, the up-regulated expression of the Cluster-2274.56424 (K13071, 1.14.15.17) gene enhances the synthesis of the chlorophyll catabolite during chlorophyll biosynthesis.

Three common differentially expressed genes of porphyrin and chlorophyll metabolism pathway with the same expression pattern (Cluster-2274.8338, Cluster-2274.38688, Cluster-2274.78784) in the variegated *Shiranuhi* and *Huangguogan* seedlings were blasted in Citrus Sinensis Annotation Project (http://citrus.hzau.edu.cn/orange/index.php). The sequence of Cluster-2274.8338 was similar to Cs6g16200 (the e-value was 4e-77, and ident was 83%), which is the ZIP1-like gen of *Citrus sinensis*. In addition, the sequences of *ZIP1* (GenBank: FJ940751.1) of *Oryza sativa* Japonica Group (Japanese rice) in NCBI have been compared with transcriptome data by sequence alignment. The percentage of identical matches between *ZIP1* and Cluster-2274.8338 was 99.92%, the total score was 2261, and the expect value (e-value) was 1.37e-26. On the other hand, Cluster-2274.8338 (*ZIP1*) was down-regulated in R_V and Y_V, and the log2 FC were −2.7399 and −2.5467, respectively (Fig. 10). Previous studies have found that *EaZIP* transcripts may not accumulate or become unstable in the early stage of plastid development^36^, leading to the loss of MPE cyclase, and the low expression results in chlorophyll losing and forming the diverse phenotype of leaf color^11^. Cluster-2274.38688 was similar to Cs8g15480.1, which encodes protochlorophyllide-dependent translocon component 52 (PTC52). PTC52 is considered to be part of a unique transposon and is most abundant in etiolated plants^37^. In this study, Cluster-2274.38688 gene was found to be up-regulated in variegated seedlings (Fig. 10). In addition, Cluster-2274.78784 was similar to Cs9g07520 (the total score was 3675, query cover was 78%, e-value was 0, and ident was 99%), which was noted to be involved in chlorophyll catabolic process (GO: 0015996). The up-regulated expression of Cluster-2274.78784 might lead to the decomposition of Chl *b* to chlorophyllide *b*. These results indicated that the formation of variegated seedlings may be caused by decreased chlorophyll synthesis and increased catabolism.

Nuclear-encoded genes affecting photosynthesis are co-expressed to assemble PSI and PSII during the de-etiolation of plants^34,38^. The transcription levels of several photosynthesis-related genes, such as *PsbA*^39^, *LHCA1–6* and *LHCB1–6*^40^, as well as *LSU* and *SSU*, are regulated by light during chloroplast biogenesis in de-etiolating plants^41^. In this study, we detected 9 common DEGs involved in the photosynthesis pathways of the variegated *Shiranuhi* and *Huangguogan* seedlings. Additionally, we identified 6 common DEGs related to porphyrin and chlorophyll metabolism. The differential expression of these genes may be critical for the leaves variegation in citrus seedlings, but need to be confirmed in future work.

## Materials and Methods

### Plant materials

**Figure 11:**
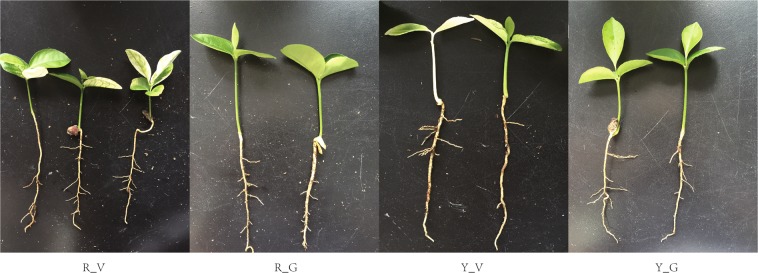
Variegated and green *Shiranuhi* and *Huangguogan* seedlings 20 days after germination. R_V, *Shiranuhi* variegated seedlings. R_G, *Shiranuhi* green seedlings. Y_V, *Huangguogan* variegated seedlings. Y_G, *Huangguogan* green seedlings.

The *Shiranuhi* and *Huangguogan* hybrid citrus cultivars analyzed in this study (Fig. 11) were provided by the Institute of Pomology and Olericulture, Sichuan Agricultural University, China. Refer to the method of Xiong *et al*.^23^ for seed germination and seedling culture. Seeds were presoaked in water for 4 h and then incubated at 25 °C for 3 days, sown in pots (vermiculite: perlite = 1:1) and then transferred to a growth chamber at 25 °C, 50–60% relative humidity, and 12-h light/12-h dark period. After germination (i.e., radicle breaks through the seed coat), seedlings were watered every 2 days. The *Shiranuhi* variegated seedlings (R_V), *Shiranuhi* green seedlings (R_G), *Huangguogan* variegated seedlings (Y_V), and *Huangguogan* green seedlings (Y_G) sample contained more than 20 seedlings were used for physiological and transcriptome analyses.

### Photosynthetic characteristics

Three seedlings of each sample were selected as test materials. The photosynthetic parameters of the second fully unfolded leaves from the base of the seedlings were recorded 20 days after germination. The Pn, Tr, Ci, and Gs were measured using the LI-6400 portable photosynthesis system (Li-Cor, Lincoln, NE, USA). The photosynthetically active radiation and CO_2_ concentration were 1,200 µmol m^−2^ s^−1^ and 400 µmol m^−2^ s^−1^, respectively. Additionally, Gs and Ci were determined at a saturated light intensity of 1,000 µmol m^−2^ s^−1^ and 70% relative humidity^42^.

### Chlorophyll and carotenoid contents

All leaves, used for the determination of photosynthetic characteristics, were harvested from variegated and green seedlings for each cultivar at 20 days after germination. Once harvested, plant materials were quick frozen using liquid nitrogen and stored at −80 °C until using. The chlorophyll and carotenoid contents of seedlings 20 days after germination were estimated as previously described using the following epuations^43^: Chl *a* = (12.7*OD*_663_ − 2.69*OD*_645_) × 10/1000 W; Chl *b* = (22.9*OD*_645_ − 4.68*OD*_663_) × 10/1000 W; Carotenoid = (1000*OD*_470_ − 3.27 Chl *a* − 104 Chl *b*)/229 × 10/1000 W.

### Root and shoot dry weights and lengths

All seedlings, used for the determination of photosynthetic characteristics, were collected and divided into shoots and roots. The root and shoot lengths were measured as previously described^44^. After the green removing process at 110 °C for 24 h, the dry weight of root and shoot was measured.

### RNA extraction and qualification

Three biological replicates were collected for *Shiranuhi* and *Huangguogan* seedlings. Total RNA was extracted from R_V, R_G, Y_V, and Y_G with the PureLink Plant RNA Reagent (Invitrogen, CA, USA). The purity of the extracted RNA was checked using the NanoPhotometer spectrophotometer (IMPLEN, CA, USA), while RNA integrity was assessed using the RNA 6000 Nano Assay Kit of the Bioanalyzer 2100 system (Agilent Technologies, CA, USA)^45^.

### Preparation of cDNA libraries and transcriptome sequencing

We purified mRNA from 1.5 µg total RNA using poly-T oligo-attached magnetic beads. The RNA was fragmented using divalent cations in 5 × NEBNext First Strand Synthesis Reaction Buffer. First-strand cDNA was synthesized using a random hexamer primer and M-MuLV Reverse Transcriptase (RNase H^−^). Second-strand cDNA was subsequently synthesized using DNA Polymerase I and RNase H^23^. The remaining overhangs were converted into blunt ends by exonuclease/polymerase activities. The NEBNext adapter with a hairpin loop structure was ligated to adenylated 3′ ends of DNA fragments for a subsequent hybridization. To preferentially select cDNA fragments that were 150–200 bp long, the library fragments were purified with the AMPure XP system (Beckman Coulter, Beverly, USA). The size-selected, adapter-ligated cDNA sequences were treated with 3 µl USER Enzyme (NEB, USA) at 37 °C for 15 min followed by a 5-min incubation at 95 °C. A PCR was then completed using Phusion High-Fidelity DNA polymerase, Universal PCR primers, and an Index (X) Primer. The PCR products were purified using the AMPure XP system, and library quality was assessed using the Bioanalyzer 2100 system^46^. The index-coded samples were clustered with the cBot Cluster Generation System of the TruSeq PE Cluster Kit v3-cBot-HS (Illumina)^47^. The resulting libraries were sequenced using the HiSeq. 4000 system (Illumina), which generated paired-end reads.

### Quality control

Refer to the previous method, raw data (raw reads) in a fastq format were first processed using in-house perl scripts. Clean data (clean reads) were obtained by removing reads containing the adapter, reads with poly-N sequences, and low quality reads from the raw data. Additionally, the Q20, Q30, GC content, and sequence duplication level were calculated for the clean reads, which were used in all downstream analyses^48^.

### Functional annotation of genes

Gene functions were annotated based on the GO^49^ and KEGG pathway databases^50^.

### Analysis of differentially expressed genes

Gene expression levels were estimated using the RSEM program^51^. Clean reads were mapped based on the assembled transcriptome, and the read count for each gene was obtained from the mapping results. Genes differentially expressed between two groups were analyzed using the DESeq R package (1.10.1), which provides statistical algorithms for detecting DEGs using a model based on a negative binomial distribution. The resulting *P* values were adjusted using the Benjamini and Hochberg’s approach to control the false discovery rate^52^. Genes with an adjusted *P* value < 0.05 following the DESeq analysis were considered to be differentially expressed between groups^52^.

### Analysis of GO and KEGG pathway enrichment of differentially expressed genes

The GO enrichment analysis of the DEGs was completed using the GOseq R packages based Wallenius’ non-central hypergeometric distribution^53^, which can adjust for gene length biases among the DEGs. We used KOBAS software to test the significance of the enrichment of DEGs in specific KEGG pathways^54^.

### Validation by qRT-PCR analysis

Leaves harvested from three independent seedlings of variegated and green samples of both *Huangguogan* and *Shiranuhi* were used as three biological replicates. Quantitative real-time PCR (qRT-PCR) analysis using the Bio-Rad CFX Manager (Bio-Rad, USA) with SsoFastTM EvaGreen Supermix (Bio-Rad) was employed to verify the DEG expression results^23^. Primers (Table 2) for specific genes encoding photosynthesis DEGs were designed using Primer3 (http://bioinfo.ut.ee/primer3-0.4.0/) and synthesized by Sangon Biotech^44^. All primers were amplified with no template control to make sure the amplicons were not primer dimers. Gene expression levels were normalized against the geometric mean of citrus reference gene, *Actin* (GenBank: XM 006480741.2) and calculated by 2^−ΔΔCT^ method^55^.

**Table 2:** Primers for qRT-PCR analysis.

Gene id	Gene Length	Primer Forward	Primer Reverse
Cluster-2274.72645	1601	TCAACCAGCCTTGTTTAGGC	CAGGCGAGATTCTGAAGACC
Cluster-2274.51835	2385	AGGCTTTTGCCTCTGTTCAA	TACACCAAAAGATGCGGTCA
Cluster-2274.81464	791	CATCTCCTTCCTTCGCACTC	ATGGTCTCATCAGGGTCCAC
Cluster-2274.57012	4559	TAACCTCCTCGGTTTTGTGG	ACCCTCCTACTCTCCCTCCA
Cluster-2274.57179	6339	TTTTGCTCACGTCTCGAATG	TATCCGGTGTGGAAGTAGGC
Cluster-2274.55964	1804	TTGATGTTCCTTTGGCTTCC	ACGGCCTCAAATTACACAGG
Cluster-2274.39503	1609	TTGTCATCGAGGAAGGAACC	TCTCCCAATTTCCAAGCATC
Cluster-2274.38688	1290	AGATCCCGCTGAATTGTGAC	GCAATTCAGTCTCCCAGAGC
Cluster-2274.56424	2492	AGATCCCGCTGAATTGTGAC	CTTTTGACGGGTGTGGATCT
Cluster-2274.78784	2700	GTATGTTGCTTTCCCCCTGA	TTGGTATGCCGGTAATGGTT
Cluster-2274.66782	752	GAAGTTGCACTCCACCCAAC	GGTGCGTACACTTGCTGAGA
Cluster-2274.70486	2669	TCCACCCAACCTTCTGTAGC	AAAGCAGATGGGATGATTGC
Cluster-2274.8338	1878	CATTCTTCTCCGGTCGTGAT	TGAAAACCTGGACAATGCAA
Actin (GenBank: XM 006480741.2)	1674	CCTCACTGAAGCACCACTCA	GTGGAAGAGCATACCCCTCA
